# Multilayer Thin-Film Optical Filters for Reflectance-Based Malaria Diagnostics

**DOI:** 10.3390/mi12080890

**Published:** 2021-07-28

**Authors:** Mariana S. Costa, Vitória Baptista, Gabriel M. Ferreira, Duarte Lima, Graça Minas, Maria Isabel Veiga, Susana O. Catarino

**Affiliations:** 1 Microelectromechanical Systems Research Unit (CMEMS-UMinho), School of Engineering, Campus de Azurém, University of Minho, 4800-058 Guimarães, Portugal; a77120@alunos.uminho.pt (M.S.C.); id8971@alunos.uminho.pt (V.B.); a81445@alunos.uminho.pt (G.M.F.); a79111@alunos.uminho.pt (D.L.); gminas@dei.uminho.pt (G.M.); 2 Life and Health Sciences Research Institute (ICVS), School of Medicine, Campus de Gualtar, University of Minho, 4710-057 Braga, Portugal; mariaveiga@med.uminho.pt; 3 ICVS/3B′s–PT Government Associate Laboratory, 4710-057 Braga/Guimarães, Portugal

**Keywords:** diagnostics, malaria, optical filters, reflectance, spectrophotometry, TFCalc

## Abstract

Malaria diagnosis relies on optical microscopy and/or rapid diagnostic tests based on detecting specific malaria antigens. The clinical sensitivity of these methods is highly dependent on parasite density, with low levels of detection at low parasite density, challenging the worldwide malaria elimination efforts. Therefore, there is a need for diagnostic methods with higher sensitivity, demanding innovative diagnostics devices able to detect malaria at low parasite density and at early stages of the disease. We propose an innovative optical device for malaria diagnosis, based on optical reflectance spectrophotometry, for the detection of parasites through the quantification of haemozoin. For this purpose, a set of eight thin-film optical filters, based on multilayer stacks of MgO/TiO_2_ and SiO_2_/TiO_2_ thin-films, with high transmittance and low full width at half maximum (FWHM) at specific wavelengths, was designed and fully characterized (both numerically and experimentally). A preliminary assessment of its potential to reconstruct the original spectra of red blood cells was performed, both in uninfected and *Plasmodium falciparum*-infected samples. The obtained results show that, although the experimental filters have a non-ideal performance characteristic, they allow us to distinguish, based on only 8 discrete points in the optical spectrum, between healthy and malaria infected samples, up to a detection limit of 12 parasites/μL of red blood cells. Those results enhance the potential of using such a device for malaria diagnostics, aiming for non-invasiveness.

## 1. Introduction

Malaria is an infectious disease and a serious public health problem in 87 countries worldwide [1,2]. According to the latest report from the World Health Organization (WHO, Geneva, Switzerland), in 2020, 229 million people were infected with malaria and from those infections resulted 409,000 deaths [2], mainly in the endemic regions with poor economic and sanitary conditions [3]. The main methods for field malaria diagnosis are based on the detection of the etiological agent, *Plasmodium* spp., in the patients′ blood through optical microscopy [4] and/or through rapid diagnostic tests (RDT) based on detecting specific malaria antigens [5]. Although these methods are low cost and relatively easy to implement, they face several challenges in many malaria-endemic regions, including the requirement for expert microscopists, inadequate quality control and the possibility of false-negative results due to low parasitaemia (<20 parasites/μL). Consequences of misdiagnosis may delay treatment, increasing the risk of disease severity and increasing the number of persons capable of infecting mosquitoes in the community. Molecular diagnosis by polymerase chain reaction (PCR) [4,6], although it allows the detection of low parasitaemia infections (down to 1–5 parasites/μL), implies sophisticated laboratories unavailable in endemic regions and highly skilled personnel and, therefore, it is recommended by the WHO not for diagnosis but only for research purposes [2]. Considering sustainable development goals, there is an urge for malaria control and the eventual elimination of the disease, demanding new technologies allowing higher sensitivity than the current methods, lower cost, non-invasiveness, easy handling, immediate feedback and environmentally more sustainable alternatives.

Malaria is a disease transmitted to human beings by the bite of the female *Anopheles* mosquito, which, when infected, has *Plasmodium* parasites in its salivary glands [7]. Within the complex life cycle of the malaria parasite, the symptomatic stage of the infection happens inside the red blood cells (RBCs), with a 48-h cycle maturation (in case of *Plasmodium falciparum*) that goes from parasite invasion of the RBC, up to RBC burst and release of new parasites ready to invade new RBCs. This stage of infection leads to a set of morphological and biochemical reactions on the RBCs when infected by the parasites. The main phenomena are the uptake and breakdown, by the parasite, of the RBC haemoglobin, as a source of nutrients, which leads to the release of the toxic haem group, that the parasite cannot digest. To detoxicate it, the parasite is capable of polymerising haem into inert crystal particles, called malaria pigment or haemozoin [8]. This process happens inside the parasite throughout its developmental stages, passing from a ring stage, trophozoite and schizont, leading to an increase in haemozoin crystals, while the haemoglobin concentration decreases throughout these stages. Although this process of haemoglobin degradation and haemozoin formation starts at the early ring stage, the major part of this process takes place in further developmental stages (trophozoite and schizont), where most of the metabolic activity happens, this being the reason why the malaria pigment is more visible under light microscopy at later stages. Haemoglobin and haemozoin present distinct optical characteristics, mainly in the visible range, with different absorbance and reflectance spectra, and those spectra diverge depending on the molecules′ concentrations in the blood [9]. As healthy human blood does not contain haemozoin in its constitution, this is a unique product that can be used as a biomarker for malaria detection [10]. Based on this principle, optical spectrophotometry has become an alternative solution for the improvement of the current malaria diagnostic methods [11,12]. With this technology, the detection could be performed for all *Plasmodium* species that infect humans, since all of them produce haemozoin during their intraerytrocytic life cycle. Nevertheless, using haemozoin as a biomarker for malaria detection can become challenging if the measurements are based on patients′ finger prick blood samples. This relates specifically to the most prevalent and deadly parasite, *P. falciparum*, that presents cytoadherence to venous endothelial cells at mature trophozoites and schizont stages, leaving only the ring stages (containing the least haemozoin) circulating in the blood stream [13]. To overcome this and increase sensitivity, technology would have to consider this limitation.

In this work, a method for optical detection of malaria based on reflectance spectrophotometry is proposed, aiming for the exemption of blood sampling and, therefore, non-invasiveness. This technique will allow the identification of malaria parasites, at all intraerythrocytic developmental stages and from all human *Plasmodium* species, through haemozoin quantification, aiming for a detection limit better than the current microscopy or RDT diagnostic methods (aiming up to 12 parasites/µL of red blood cells) [14]. For the implementation of such a technique in a full integrated system, it will be expected that the detection system comprises a light source covering the optical visible spectrum (from 400 nm to 800 nm [9]), optical detectors (as photodiodes, that capture the reflected light and convert it into electric currents) and optical bandpass filters, tuned at specific wavelengths, with high transmittance and low full width at half maximum (FWHM). These optical filters, which are the focus of this work, allow the selection of specific wavelengths at the photodetector site, to measure and reconstruct the samples′ spectra based on a limited and discrete number of spectral bands (eight, as will be described). The filters will be based on multilayer thin-films, with a Fabry–Perot interferometer structure, compatible with CMOS (Complementary metal–oxide–semiconductor) processes. Therefore, the main goal of this study is the numerical and experimental characterization of the optical response of eight bandpass thin-film optical filters, as well as the assessment of their potential to reconstruct, based on only eight discrete wavelengths, the original continuous spectra of uninfected and infected RBCs, aiming for their integration on a malaria diagnostic device.

## 2. Methods

### 2.1. Optical Reflectance Spectra of Uninfected and Parasite Infected RBCs

**Figure 1:**
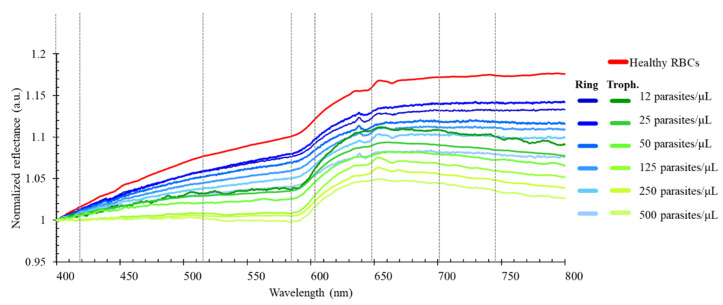
Normalized reflectance spectra (a.u.) of uninfected RBCs (in red) and RBCs with early (rings, in blue) and late (trophozoites, in green) parasites, with different *P. falciparum* parasitaemia, from 12 to 500 parasites/μL of RBCs, in the 400–800 nm range of the optical spectrum (*n* = 3) [9]. The vertical dashed lines represent the 8 selected spectral bands.

In order to select the target wavelengths for the thin-film optical filters, preliminary assays were performed to characterize the optical spectra of *P. falciparum*-infected RBCs. Figure 1 presents the average (*n* = 3) continuous normalized reflectance spectra of uninfected and cultured *P. falciparum*-infected RBCs, at different parasitaemia, from 12 to 500 parasites/μL of RBCs, both in early (rings) and late (trophozoites) stages [9]. Samples with 40% haematocrit were considered to represent the quantity of RBCs in human whole blood. Healthy human whole blood, needed for the samples′ testing and *P. falciparum* in vitro cultures, was provided by Instituto Português do Sangue e Transplantação (IPST; Portuguese Blood and Transplantation Institute), Porto, Portugal. All procedures for blood collection, transport and in vitro experiments were carried out in compliance with the EU directives 2004/23/CE, 2006/17/CE and 2006/86/CE. The spectra were measured with a spectrophotometric top-bench setup, comprising a 200 W Quartz Tungsten Halogen light source (model 66881, Oriel Newport), optical-fibre reflectance probes, a cuvette sample holder and an AvaSpec-ULS2048XL EVO spectrophotometer with an integrated monochromator (Avantes, NS Apeldoorn, The Netherlands). Barium sulphate was used as a reference for reflectance measurements. The samples were measured in 1 mm optical path cuvettes. Reflectance data were collected and post-processed using the AvaSoft 8.11 software.

From the presented spectra, we selected a set of 8 specific relevant spectral bands, in the visible range of the optical spectrum—397 nm, 419 nm, 516 nm, 585 nm, 603 nm, 649 nm, 701 nm and 746 nm—for the simulation and experimental characterization of thin-film optical filters. These 8 spectral ranges were selected as they are fairly distributed within the entire spectra and capture the main oscillations of the samples′ spectra, as previously presented. Aiming for the future integration of the thin-film optical filters in a malaria diagnostic device, and in order to allow the specific deposition of each of the filters on top of a photodiode array, the selection of 8 spectral bands is justified by a reduction of the size and complexity of the final system (when compared with a previous study of the team considering 16 spectral bands) [14], assuring the essential compromise between the accuracy of the extraction of the optical spectra and the number of wavelengths (and consequent required optical filters).

### 2.2. Design and Simulation of the Thin-Film Optical Filters

**Figure 2:**
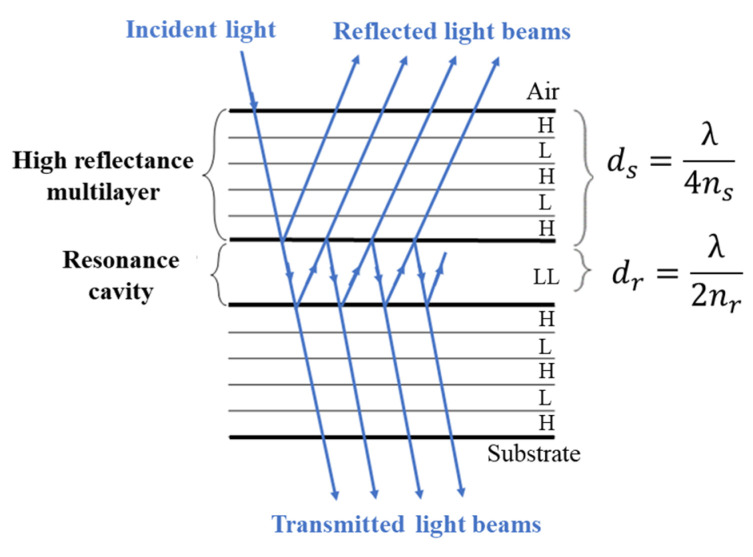
Multilayer thin-film optical filter structure. In the scheme, *λ* is the transmitted wavelength and *n* is the refractive index of the resonant cavity material. Considering a first order interference and a light incidence angle of 0°, *d_r_* and *d_s_* expressions describe the calculus of the resonance cavity and mirror thicknesses, respectively [17].

As referred to in the previous Section 2.1, the set of 8 spectral bands was selected to help to accurately extract the relevant information of the optical spectra and reconstruct the continuous reflectance spectra of the uninfected and malaria infected samples, based on a minimum number of wavelengths. The bandpass thin-film optical filters are based on multilayer structures, forming a Fabry–Perot interferometer structure, with two flat parallel mirrors separated by a resonance cavity layer, with a pre-defined thickness [15,16], as schematically represented in Figure 2.

According to the literature, a Fabry–Perot interferometer structure with dielectric mirrors usually contains 9 or more thin-film layers, this number being a compromise between low FWHM, high transmittance and fabrication constraints [18]. After preliminary simulations, aiming for a better filter performance, 11 layers per filter structure [15], comprised of five dielectric layers in each mirror with, alternately, high (H) and low (L) refractive index materials, were chosen. For each filter, the resonance cavity is characterized by a multiple-beam interference, causing a high optical transmission at a narrow band of wavelengths, around a wavelength for which the cavity is a multiple of one-half wavelength thick [17]. Therefore, assuming similar films on both mirrors, the thickness of the resonance cavity determines the tuned wavelength of the filter. Each of these mirrors is composed by dielectric films, characterized by high transmittance at specific wavelengths and low energy absorption rates. Magnesium oxide (MgO) and silicon dioxide (SiO_2_), with low refractive indexes, and titanium dioxide (TiO_2_), with a high refractive index, were selected as the dielectric materials for the thin-films’ filters, since they are rigid, extremely difficult to remove from the substrate, compatible with CMOS fabrication and their deposition processes are well characterized and documented [17].

The design and computational simulation of the 8 filters (centred at the 397, 419, 516, 585, 603, 649, 701 and 746 nm spectral bands in the visible spectrum) was performed in TFCalc 3.5 (Software Spectra Inc., Portland, OR, USA), a software tool based on finite element methods. The filters were characterized regarding their transmittance peak and FWHM, aiming for transmittance as high as possible (with at least twice the intensity of any noise peak in the considered spectral range) and FWHM around 10 nm, which can be considered acceptable for the intended application, as it avoids superposition between the spectral bands of neighbouring filters.

The optical filters were divided into three spectral regions: UV/VIS (397–419 nm), VIS (516–603 nm) and VIS/IR (649–746 nm), in order to optimize the design and fabrication processes (allowing simultaneous depositions). Besides the thickness and the properties of the films′ materials, in particular their refractive indexes (obtained from the *refractiveindex.info* database), the simulation also took into consideration the properties of the substrate (glass), incident and exit medium (air) and reference wavelength, which have influence on the filters′ optical responses. For each of the three spectral regions, a reference wavelength was selected (420 nm, 550 nm and 680 nm, respectively). Table 1 presents the combination of the layers’ thickness values for each optical filter, designed using TFCalc, and optimized (through iterative adjustments) for the highest transmittance.

As observed in Table 1, the narrow optical filters were divided into four spectral regions: 397 nm, 419 nm, 516–603 nm and 649–746 nm. For each group, the two mirrors are symmetrical and consist of five alternate layers of TiO_2_ and MgO thin-films, for the filter centred at 397 nm, and TiO_2_ and SiO_2_ thin-films, for the other seven filters. During the simulations, MgO led to a better filter performance near the UV region, when compared to SiO_2_ and, therefore, it was the selected material for the 397 nm filter. For each filter inside the spectral region, the layers of the same material have the same thickness. This process means that the definition of each spectral band can be performed only by adjusting the thickness of the MgO or SiO_2_ resonant cavities.

### 2.3. Experimental Setup

**Figure 3:**
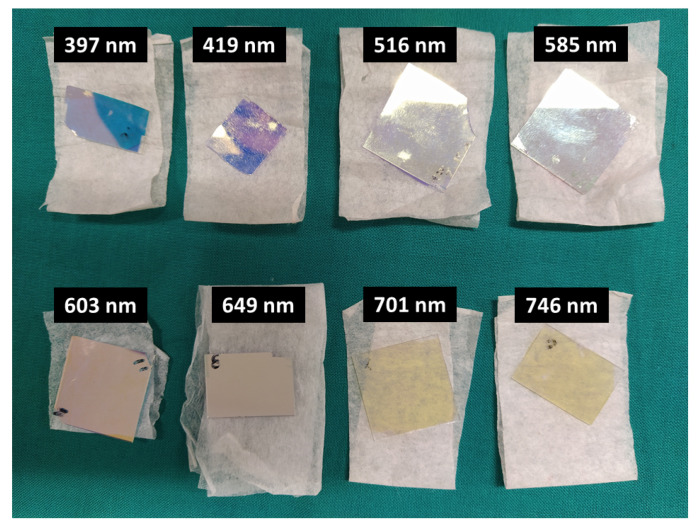
Photographs of the 8 thin-film optical filters experimentally characterized, centred at 397, 419, 516, 585, 603, 649, 701 and 746 nm, used in the experimental assays. In some of the filters, it is clear that some damage of the thin-film surfaces occurred after their prolonged use.

The proposed and simulated eight thin-film optical filters (397 nm, 419 nm, 516 nm, 585 nm, 603 nm, 649 nm, 701 nm and 746 nm) were fabricated through ion beam deposition in glass substrates and characterized in the CMEMS-UMinho laboratories, regarding their optical transmittance and FWHM. The fabrication process of the optical filters is detailed in [17,19], and it is not the focus of this study. However, it is relevant to note that, after fabrication, the optical filters may not assure the exact same optical properties as the simulated ones, as the deposition techniques and setups may lead to slight variations in the materials′ refractive indexes. To perform the experimental characterization of the fabricated optical filters, a set of long pass and short pass commercial optical filters (acquired from Edmund Optics and Thorlabs) was also used, in addition to the thin-film bandpass filters, in order to remove additional transmittance regions and noise signals that could appear outside the range of interest. Figure 3 presents photographs of the eight thin-film bandpass optical filters, centered at 397, 419, 516, 585, 603, 649, 701 and 746 nm. Figure S1 (Supplementary Material) displays examples of commercial long pass (370 nm, 550 nm and 600 nm) and short pass (500 nm) filters, also used in the experimental assays.

**Figure 4:**
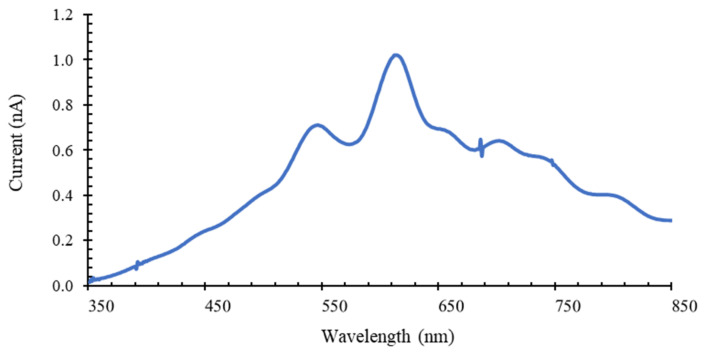
White light spectral current (A), emitted by the 200 W Quartz Tungsten Halogen light source and measured by a commercial photodiode, as function of wavelength (nm).

The optical transmittance of the eight bandpass thin-film optical filters was performed in a top bench setup at CMEMS. The setup includes a 200 W Quartz Tungsten Halogen light source (model 66881, Oriel Newport, Irvine, CA, USA), a monochromator (model 74000, Thermo Oriel instruments, Newport, Irvine, CA, USA) that selects the emission wavelength, an optical fiber transmittance probe, a mechanical support for the positioning and alignment of the optical filters, a commercial photodiode (aligned with the filters and the fiber) that converts the light transmitted at each wavelength into an electric current, a picometer (Keithley 487, Cleveland, OH, USA) to measure the current and a computer for data acquisition and post-processing using a LabView custom made tool. Figure 4 presents the incident white light spectral photocurrent as a function of wavelength, which is considered the reference baseline for the optical filters′ transmittance measurements. Therefore, for each measurement, the obtained photocurrent at each wavelength (from 350 to 850 nm) is divided by that reference curve and the transmittance spectrum is obtained.

## 3. Results

### 3.1. Optical Filters Characterization

**Figure 5:**
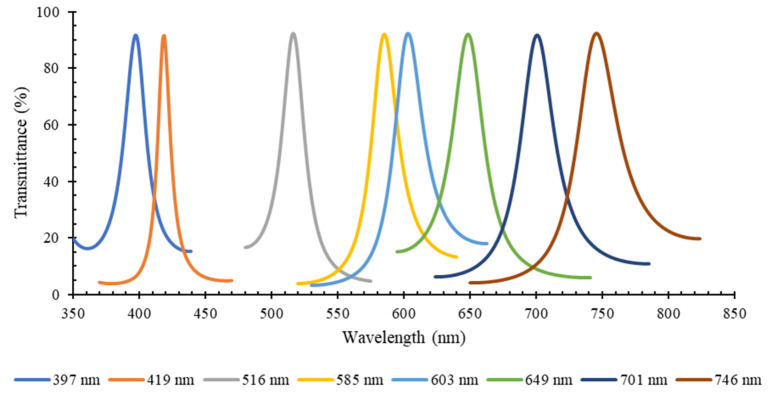
Simulation results of the central spectrum transmittance (%) vs. wavelength (nm), for the 8 designed MgO/TiO_2_ and SiO_2_/TiO_2_ optical filters, according to the structure and dimensions presented in Table 1.

Figure 5 presents the simulation of the central spectrum transmittance for the eight bandpass thin-film optical filters previously designed, according to Table 1. According to the results, in their specific spectral band, the designed filters have high transmittance (above 90% for all).

Although the FWHM results show some superposition between the 585 and 603 nm wavelength bandpass filters, these multilayer stacks, comprised of 11 layers of MgO/TiO_2_ and SiO_2_/TiO_2_ thin-films and MgO or SiO_2_ resonance cavity layers, assure relatively good optical features. Although the global performance of the optical filters could be theoretically improved by increasing the number of layers to 13, the complexity of the fabrication process would also increase.

**Figure 6:**
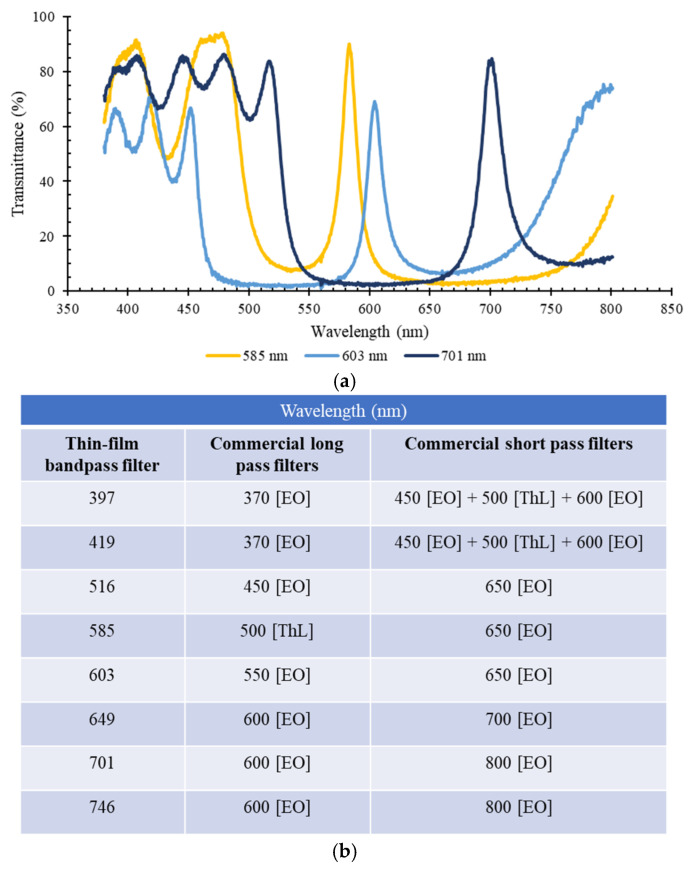
(**a**) Experimental results (*n* = 3) of transmittance (%) vs. wavelength (nm), using a spectrophotometric top bench setup for 3 of the SiO_2_ /TiO_2_ optical filters centred at 585, 603 and 701 nm, without using commercial filters; (**b**) list of the commercial long pass and short pass optical filters and respective manufacturers (EO–Edmund Optics; ThL-Thorlabs) used in combination with each of the 8 bandpass thin-film optical filters (see Figure S2 of the Supplementary Material for the optical transmittance spectra of these filters).

After the design, simulation and fabrication of the eight optical filters, they were experimentally characterized using the previously described top bench setup of Section 2.3. Figure 6a presents, for exemplification purposes, the full transmittance spectra of three of the fabricated thin-film optical filters, for the 585, 603 and 701 nm spectral bands, measured in the 350–850 nm range of the optical spectrum. For all the filters, although there is a high transmittance peak near the expected wavelength (with values always > 70%), it is inferior to the simulated one (as presented in Figure 5). Additionally, in Figure 6a, the high transmittance regions outside the relevant spectral bands are visible. These bands will be experimentally removed by superposing commercial long pass and short pass optical filters on top of the thin-film bandpass filters. This behaviour was observed for all the eight thin-film optical filters. Therefore, Figure 6b presents a list of commercial short and long pass optical filters, which were added to remove the undesired spectral bands in each of the eight targeted bandpass wavelengths. The transmittance spectra of all the commercial short and long pass filters listed in Figure 6b are presented in Figure S2 of the Supplementary Material.

**Figure 7:**
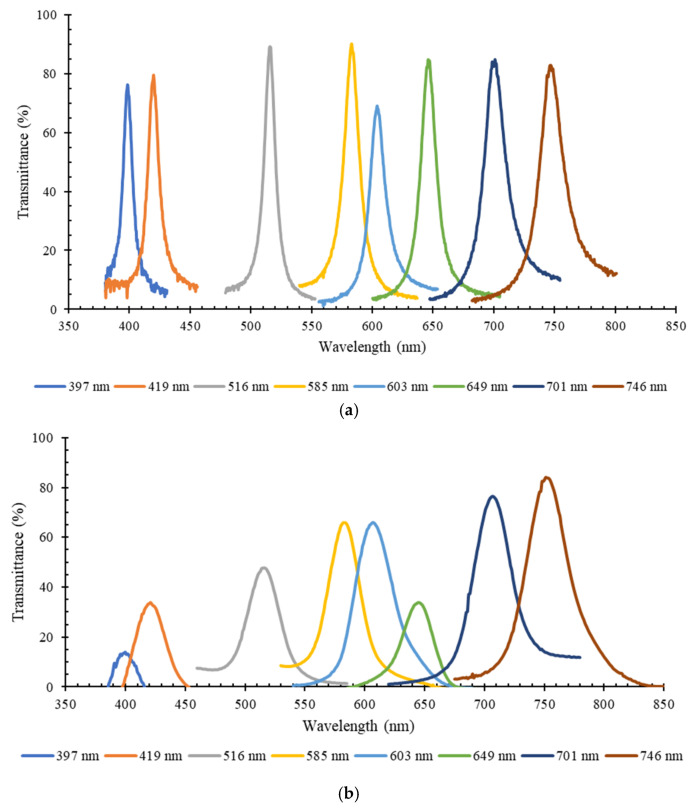
Experimental results (*n* = 3) of transmittance (%) vs. wavelength (nm), for the 8 MgO/TiO_2_ and SiO_2_/TiO_2_ optical filters, measured with a spectrophotometric top bench setup: (**a**) without using additional commercial filters; (**b**) using commercial short pass and long pass filters to remove high transmittance components outside the relevant spectral bands.

In order to assess the effect of the additional commercial long pass and short pass optical filters in the transmittance spectra of the thin-film optical filters, Figure 7 presents the experimental results of the central spectra from the peak transmittance spectra of the eight bandpass thin-film optical filters, without using any other optical filters to limit the spectral range outside the main peak region (Figure 7a); and using the different combinations of commercial long pass and short pass optical filters, as presented in Figure 6b (Figure 7b). In both cases, the transmittance peaks are clearly visible in their specific spectral bands. However, as expected, when additional filters are included (Figure 7b), the transmittance of the bandpass optical filters significantly decreases according to the combination of the transmittance of the passband commercial filters. All transmittance curves correspond to the average of three measurements.

**Figure 8:**
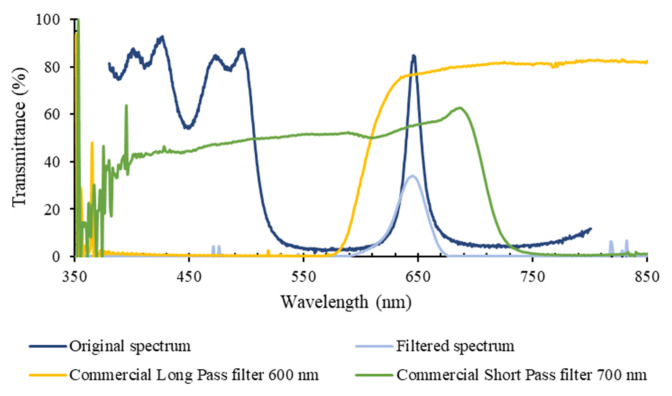
Experimental transmittance (%) vs. wavelength (nm) spectrum (*n* = 3), of the 649 nm bandpass filter, measured with the spectrophotometric top bench setup: without using additional commercial filters (dark blue line); using the commercial short pass and long pass filters (light blue); and spectra of the commercial long pass (yellow) and short pass (green) filters, used to remove high transmittance components outside the relevant spectral bands (as listed in Figure 6b).

When compared to the numerical results, the peak transmittance of the eight optical filters, in the absence of additional commercial short and long pass filters, is still high (above 70% for all the fabricated filters). After adding the commercial long and short pass filters, it was observed that almost all optical filters (except the 746 nm filter) present optical transmission values significantly lower than the simulated ones, which result from the combinations of the different required filters. Particularly, two filters closer to the ultraviolet (UV) region of the spectra (397 and 419 nm) have transmittance below 40%. On the contrary, near the infrared (IR) region, the optical filters (701 and 746 nm) present considerably good transmittance spectra, around or above 80%. For example, for the 649 nm bandpass filter, the significant reduction in the transmittance is explained by the effect of both commercial short pass and long pass filters, as shown in Figure 8. In addition to the 600 nm cutoff long pass filter, needed to remove the high transmittance of the filter in the 350–500 nm region, a 700 nm cutoff short pass filter was also needed to reduce the transmittance effect that is visibly increasing after 750 nm, and still affects the photodiode readout. As seen in Figure 8, this short pass filter has transmittance around 50% in the visible range of the spectrum, which causes the significant decrease in transmittance in the 649 nm relevant wavelength.

**Figure 9:**
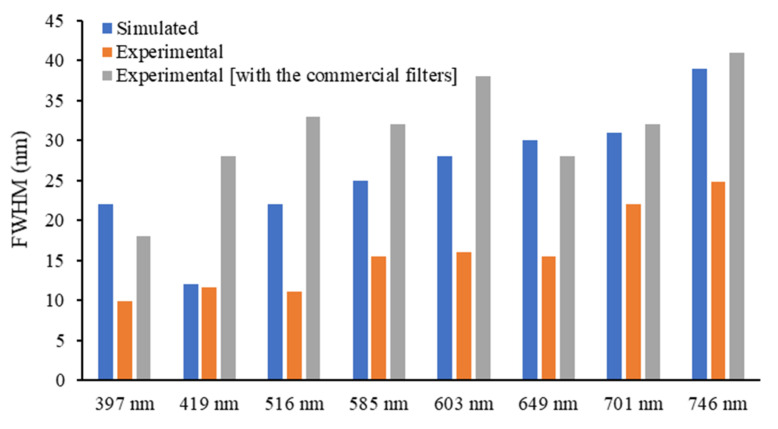
Comparison between the numerical simulated and experimental (*n* = 3) FWHM (nm) for each of the 8 MgO/TiO_2_ and SiO_2_/TiO_2_ optical filters.

Thus, these results show that, although adding short and long pass optical filters helps to reduce the interference of other optical regions in the obtained spectra, they have a significant effect in the transmittance performance of the thin-film optical filters. Regarding FWHM, it is higher than 10 nm (value assumed as ideal for the intended application) for all the optical filters′ spectra, and some superposition was observed between the spectra of the 585 nm, 603 nm and 649 nm optical filters. Figure 9 presents a comparison between the numerical and experimental FWHM, obtained for each of the eight optical filters (with and without the additional commercial short and long pass filters). The experimental data in the plot were obtained from Figure 7a,b, and the numerical data were acquired from Figure 5. It is possible to observe that all optical filters have a large FWHM (above 10 nm for all the designed filters), and it is significantly increased by adding the additional optical layers of the commercial filters, which may reduce the wavelength selectivity of the optical reading.

### 3.2. Discrete Reconstruction of the Samples’ Original Spectra: Application to Malaria Detection

**Figure 10:**
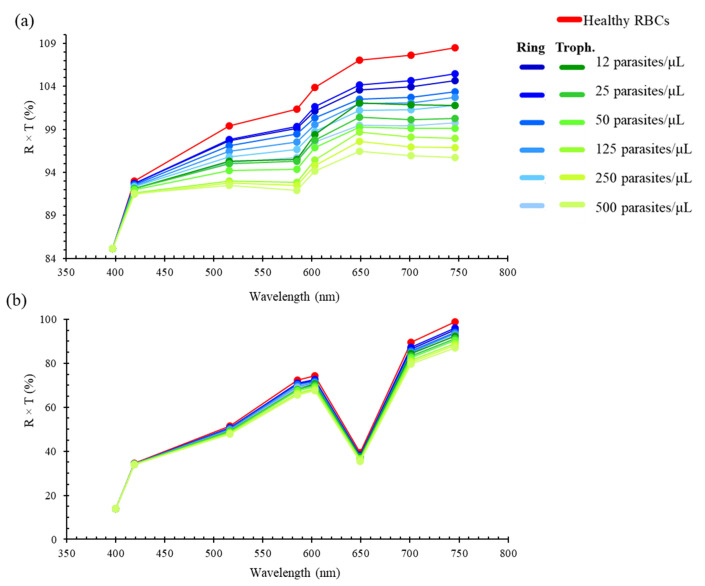
Reconstructed normalized optical reflectance spectra (%), based on 8 discrete wavelengths (represented by points in the plots), obtained from the combination between the transmittance spectra of the thin-film optical filters and the reflectance spectra of uninfected (red) and *P. falciparum*-infected RBCs samples (with different parasitaemia values, ranging from 12 to 500 parasites/μL of RBCs), in both early (blue, rings) and late (green, trophozoites) stages. (**a**) Numerical simulation; (**b**) experimental results.

After the optical characterization of the optical filters, the filters were evaluated to assess if the eight discrete spectral bands allowed them to: (1) accurately extract the optical data and reconstruct optical reflectance spectra representative of the original continuous ones (as in the previous spectra from Figure 1 and Figure 2); (2) detect differences between the optical reflectance spectra of uninfected and *P. falciparum*-infected RBCs, at different parasitaemia, for malaria diagnosis applications. For that purpose, the transmittance data of the designed eight optical filters were combined with uninfected and infected RBCs reflectance spectra, at different parasitaemia and developmental stages (rings and trophozoites), from 12 to 500 parasites/μL of RBCs. Figure 10 shows the resultant discrete spectra, based on eight spectral bands, obtained both numerically and experimentally (in the latter, with the addition of the commercial filters according to table of Figure 6b, assuring that only the high transmittance peak of the thin-film bandpass filters was considered).

**Figure 11:**
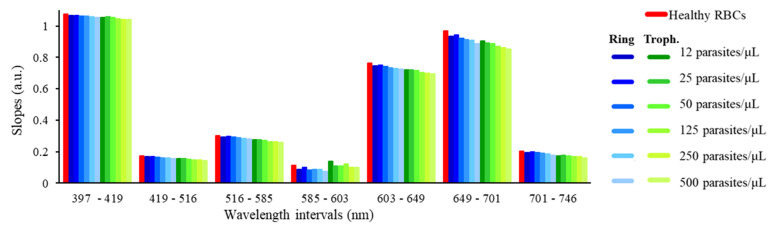
Bar plots representing the slopes of the lines between the experimental reconstructed reflectance spectra (%) at different wavelengths (397–419 nm, 419–516 nm, 516–585 nm, 585–603 nm, 603–649 nm, 649–701 nm, 701–746 nm), for uninfected (red) and *P. falciparum*-infected RBCs samples (with different parasitaemia values, between 12 and 500 parasites/μL of RBCs), in both early (blue, rings) and late (green, trophozoites) developmental stages.

The results show that, with the numerically simulated optical filters, it is possible to correctly extract the original reflectance spectra (Figure 1), based on only eight spectral bands. The experimental assays showed that the optical filters failed to correctly reconstruct the 649 nm region of the original spectra, with a clear decrease at that spectral band, when compared to the original continuous spectra. That variation is mainly explained by the significantly lower transmittance of the 649 nm optical filter (when the commercial short and long pass optical filters are also considered, as seen in Figure 7b and Figure 8. However, since that trend is the same in all samples, the relative oscillations between different optical filters can be easily removed in a postprocessing phase, through an additional calibration or normalization step. Based on the experimental results of Figure 10b, it is observed that, between 397 nm and 516 nm, there is no visible difference between uninfected and infected RBCs. However, above 516 nm, there is an increase in the resultant spectra up to 600 nm, with slopes becoming higher as the parasitaemia decreases, between the several analysed spectral bands. It is also possible to distinguish between uninfected (red) and infected RBCs when the parasites are at different developmental stages: ring (blue) and trophozoite (green) stage. In order to evaluate the possibility of implementing simple detection and diagnostics algorithms, based on the data from the reflectance spectra, the slopes between the reflectance values at different wavelengths of the spectra were calculated through the (y_2_ − y_1_)/(x_2_ − x_1_) expression. Figure 11 presents a selection of calculated slopes, between the reconstructed reflectance values at different wavelengths, obtained from the experimentally reconstructed spectra shown in Figure 10b. Table 2 presents the variation of the slopes (%) obtained for each *P. falciparum*-infected RBCs sample (with different parasitaemia values, between 12 and 500 parasites/μL of RBCs), when compared to the uninfected RBCs, for the different wavelength intervals under analysis. The plot shows that, for a set of wavelength ranges, there is a variation in the slope as the parasitaemia varies in the RBCs samples. Particularly, in the experimental results, as the parasitaemia increases, the slopes clearly decrease, and this variation is higher at the 603–649 nm and 649–701 nm ranges, where different samples can be more easily distinguished. The results allow us to predict that, from a reduced number of optical filters (only eight spectral bands), it is possible to partially reconstruct the optical reflectance spectra of uninfected and infected RBCs samples with different parasitaemia, showing high potential for the intended application.

The analysis of Table 2 shows that, for most wavelength ranges, while for low parasitaemia the variation of the slopes between the uninfected and infected samples may be difficult to detect, it is clearly higher as the parasitaemia increases. The exception is the 585–603 nm range, where no trend is observed. Additionally, from Table 2, it is possible to evaluate the best wavelength intervals to consider for an accurate decision algorithm. For example, while the 397–419 nm interval results in a low variation in the slopes (below 3% for all parasitaemia and parasite stages), the wavelength intervals 516–585 nm and 701–746 nm show that, for 500 trophozoites/uL, the slopes vary up to more than 15% and 20%, respectively, when compared to the uninfected RBCs. At these wavelength ranges, even for the lower parasitaemia values (12 parasites/μL in the ring stage), the variation is higher than 3% (which was the maximum variation detected at the 397–419 nm range). The lower variation at the 397–419 nm interval is probably due to the lower transmittance of the filters at those wavelengths, which leads to a decrease in the performance of the system in those spectral ranges. These results show that the transmittance of the filters, resultant from the superposition of the thin-film optical filters and the commercial ones, has a significant impact on the distinction between the *P. falciparum*-infected and uninfected samples. Additionally, as the FWHM of the optical filters is high (around 30 nm, as experimentally measured), it means that the filters are not specific for a single or highly narrow wavelength.

Despite the limitations of the method, concerning the low transmittance and high FWHM of the filters and the consequent reduced slope variations that were observed for some wavelength ranges, and which may lead to a minor resolution of the detection, it is possible to infer that the system, with the inclusion of a high precision decision algorithm based on line slopes, may be able to distinguish between uninfected and infected RBCs samples, enhancing its potential as a malaria diagnostic tool. It is relevant to notice that a decision algorithm must take into account the maximum possible information, by combining different wavelength ranges, in order to increase, as much as possible, the sensitivity and specificity of the algorithm.

## 4. Conclusions

This paper presented the design, simulation, experimental characterization and viability of eight narrow bandpass MgO/TiO_2_ and SiO_2_/TiO_2_ thin-film optical filters, for the development of an optical reflectance-based malaria diagnostic device. As the deposition techniques and experimental setups may lead to slight variations in the materials′ refractive indexes, the fabricated optical filters may not assure the exact same optical properties as the simulated ones. Thus, to compare the optical features of the filters, they were numerically and experimentally characterized regarding their optical transmittance and FWHM. While in the numerical simulations all the optical filters had transmittance above 90%, in the experimental results the transmittance values (after including the commercial short and long pass filters to remove unwanted transmittance peaks outside the relevant spectral range) were between approximately 18% (minimum, at 397 nm) and 85% (maximum, at 746 nm). The FWHM values, for both experimental and simulated optical filters, were above 10 nm, showing some superposition of the spectra between the 585 and 603 nm spectral bands. Further improvements need to be addressed in the fabrication of the thin-film optical filters, in order to reduce the high transmittance regions outside the targeted wavelength. Such improvements will lead to the reduction of the number of commercial short and long pass filters that are needed to remove the transmittance outside the targeted spectral bands, thus improving the transmittance of the filters and simplifying the final device. Despite some deviations in the transmittance spectra between the simulated and experimental filters, the eight fabricated bandpass optical filters showed success in extracting the optical information and reconstructing the reflectance spectra of uninfected and *P. falciparum*-infected RBCs. The system, based on only eight spectral bands, was able to distinguish them up to a detection limit of 12 parasites/μL of RBCs, even just considering parasites at early ring stage, which revealed superior sensitivity when compared with the currently available diagnostic methods in the field (RDT and microscopy). These results are an improvement towards point-of-care single chip fabrication, in comparison to the previous study with 16 wavelengths [14], showing that even with less spectral bands and a consequent simpler fabrication process, it is still possible to detect parasitaemia variations.

## Figures and Tables

**Table 1:** Optical filters in the UV/Vis, Vis and Vis/IR spectral regions and respective MgO/TiO_2_ and SiO_2_/TiO_2_ layer thicknesses (RC: Resonance Cavity).

Maximum Transmittance Peaks Per λ (nm)									
	397		419	516	585	603	649	701	746
**Thickness layer (nm)**									
TiO_2_	40	TiO_2_	44	63			80		
MgO	63	SiO_2_	75	97			118		
TiO_2_	40	TiO_2_	44	63			80		
MgO	63	SiO_2_	75	97			118		
TiO_2_	40	TiO_2_	44	63			80		
MgO (RC)	110	SiO_2_ (RC)	141	150	218	236	198	249	294
TiO_2_	40	TiO_2_	44	63			80		
MgO	63	SiO_2_	75	97			118		
TiO_2_	40	TiO_2_	44	63			80		
MgO	63	SiO_2_	75	97			118		
TiO_2_	40	TiO_2_	44	63			80		

**Table 2:** Variation in the slopes (%) obtained for each *P. falciparum*-infected RBCs sample (with different parasitaemia values, between 12 and 500 parasites/μL of RBCs), when compared to the uninfected RBCs, for the different wavelength intervals: 397–419 nm, 419–516 nm, 516–585 nm, 585–603 nm, 603–649 nm, 649–701 nm, 701–746 nm.

	Slope Variation (%)											
	Rings (Parasites/μL)						Trophozoites (Parasites/μL)					
**Wavelength Interval (nm)**	**12**	**25**	**50**	**125**	**250**	**500**	**12**	**25**	**50**	**125**	**250**	**500**
397–419	0.5	0.5	1.0	1.0	1.3	1.8	2.0	1.6	1.9	2.9	3.0	2.9
419–516	4.5	4.3	5.9	7.8	9.4	10.7	10.2	11.5	13.6	16.0	16.6	17.6
516–585	3.5	2.7	4.0	5.6	6.8	8.4	9.8	9.6	10.6	13.2	13.6	15.1
585–603	22.8	12.0	26.9	22.9	24.1	32.7	−22.7	2.3	5.4	−5.9	11.1	9.6
603–649	2.4	1.8	2.8	3.9	4.7	5.3	5.7	5.7	6.5	8.3	8.7	9.0
649–701	3.7	2.9	5.0	5.6	6.4	8.4	6.7	7.9	8.7	10.1	11.1	12.2
701–746	4.9	3.5	5.4	7.0	8.9	11.9	14.2	12.9	14.3	16.8	17.9	20.8

## Data Availability

All data are available upon request to the corresponding author.

## Supplementary Materials

The following are available online at https://www.mdpi.com/article/10.3390/mi12080890/s1, Figure S1: Photographs of examples of the commercial long pass (370 nm, 550 nm and 600 nm) and short pass (500 nm) filters, used in the experimental assays; Figure S2: Spectra of the commercial long (**a**) and short (**b**) pass optical filters used in the experimental setup and listed in Figure 6b.
