# Supplementary material for: Multilayer Thin-Film Optical Filters for Reflectance-Based Malaria Diagnostics
Source: Micromachines (Basel). 2021 Jul 28;12(8):890. doi: 10.3390/mi12080890 (PMC8398211; doi:10.3390/mi12080890)
Supplement: Supplementary file 1 [file micromachines-12-00890-s001.zip › micromachines-1314300-supplementary.pdf]

Supplementary material

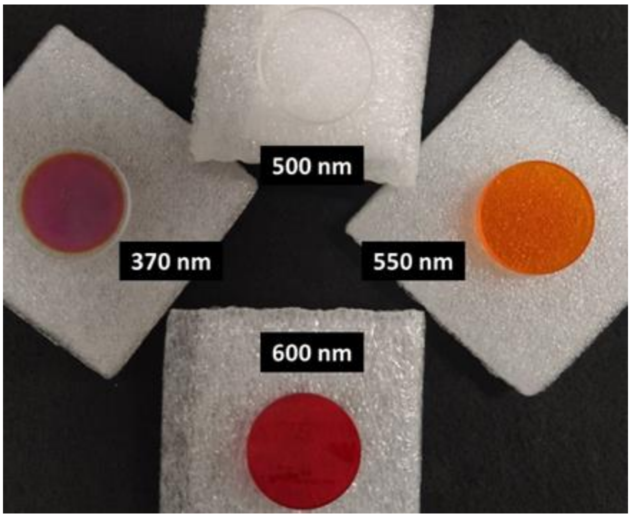

Figure S1: Photographs of examples of the commercial long pass (370 nm, 550 nm and 600 nm) and short pass (500 nm) filters, used in the experimental assays.

(a)

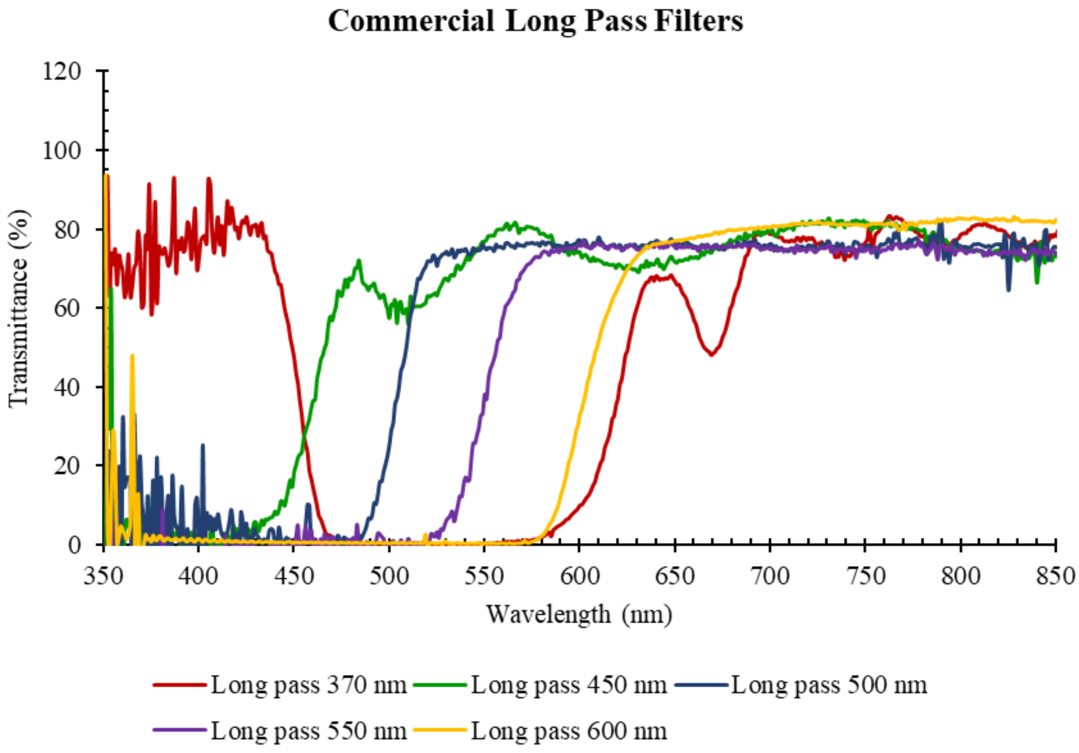

(b)

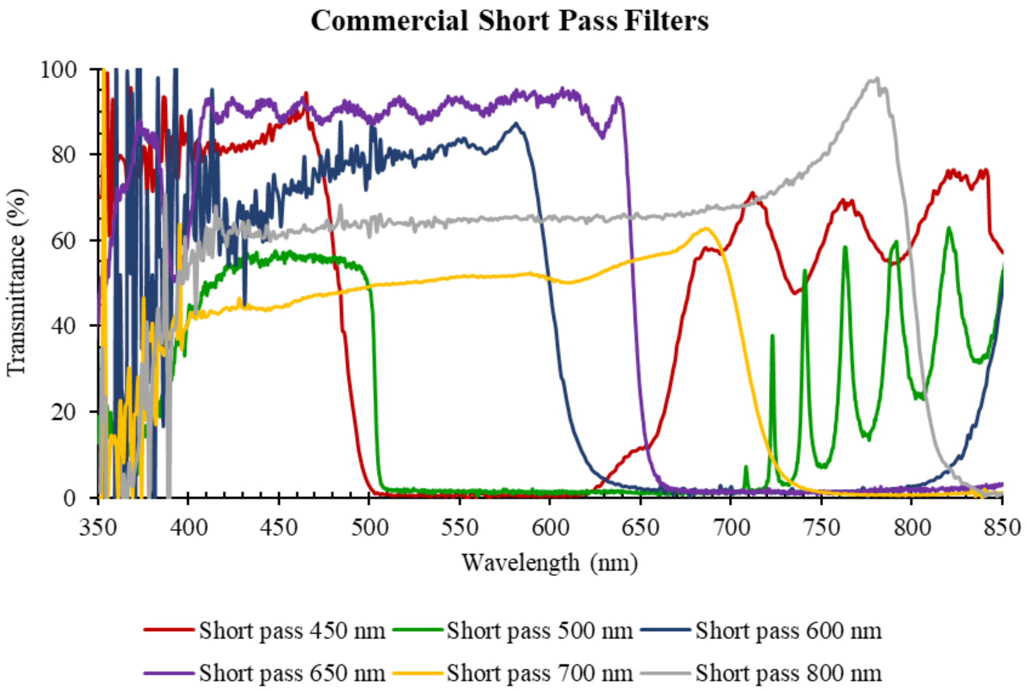

Figure S2: Spectra of the commercial long (a) and short (b) pass optical filters used in the experimental setup and listed in Figure 6 (b).
